# *“Community members have more impact on their neighbors than celebrities”*: leveraging community partnerships to build COVID-19 vaccine confidence

**DOI:** 10.1186/s12889-023-15198-6

**Published:** 2023-02-16

**Authors:** Maria Tjilos, Autumn L. Tamlyn, Elizabeth J. Ragan, Sabrina A. Assoumou, Katherine Gergen Barnett, Petrina Martin, Rebecca B. Perkins, Benjamin P. Linas, Mari-Lynn Drainoni

**Affiliations:** 1 Section of Infectious Diseases, Boston Medical Center, 801 Massachusetts Ave. Crosstown Center, 2nd Floor, 02118 Boston, MA US; 2 Section of Infectious Diseases, Boston University Chobanian & Edward Avedisian School of Medicine, Boston Medical Center, 72 E Concord St, 02118 Boston, MA US; 3 Department of Family Medicine, Boston Medical Center, 1 Boston Medical Center Place, 02118 Boston, MA US; 4 Boston Medical Center, Boston Medical Center Health System, 85 East Concord Street, 02118 Boston, MA US; 5 Department of Obstetrics and Gynecology, Boston University Chobanian & Edward Avedisian School of Medicine, 72 E Concord St, 02118 Boston, MA US; 6 Department of Epidemiology, Boston University School of Public Health, 715 Albany St, 02118 Boston, MA US; 7 Department of Family Medicine, Boston University Chobanian & Edward Avedisian School of Medicine, 72 E Concord St, MA 02118 Boston, United States; 8 Harvard Center for Primary Care, Harvard Medical School, 25 Shattuck St, MA 02115 Boston, US; 9 Department of Obstetrics and Gynecology, Boston Medical Center, 775 Albany St, MA 02118 Boston, US; 10 Department of Health Law, Policy & Management, Boston University School of Public Health, 715 Albany St, MA 02118 Boston, US

**Keywords:** Community engagement, COVID-19, Vaccine confidence, Public health messaging

## Abstract

**Background:**

Vaccines are a strong public health tool to protect against severe disease, hospitalization, and death from COVID-19. Still, inequities in COVID-19 vaccination rates and health outcomes continue to exist among Black and Latino populations. Boston Medical Center (BMC) has played a significant role in vaccinating medically underserved populations, and organized a series of community-engaged conversations to better understand community concerns regarding the COVID-19 vaccine. This paper describes the themes which resulted from these community-engaged conversations and proposes next steps for healthcare leaders.

**Methods:**

We accessed nine publicly available recordings of the community-engaged conversations which were held between March 2021 and September 2021 and ranged from 8 to 122 attendees. Six conversations prioritized specific groups: the Haitian-Creole community, the Cape Verdean community, the Latino community, the Black Christian Faith community, guardians who care for children living with disabilities, and individuals affected by systemic lupus erythematosus. Remaining conversations targeted the general public of the Greater Boston Area. We employed a Consolidated Framework for Implementation Research-driven codebook to code our data. Our analysis utilized a modified version of qualitative rapid analysis methods.

**Results:**

Five main themes emerged from these community-engaged conversations: (1) Structural factors are important barriers to COVID-19 vaccination; (2) Mistrust exists due to the negative impact of systemic oppression and perceived motivation of the government; (3) There is a desire to learn more about biological and clinical characteristics of the COVID-19 vaccine as well as the practical implications of being vaccinated; (4) Community leaders emphasize community engagement for delivering COVID-19 information and education and; (5) Community leaders believe that the COVID-19 vaccine is a solution to address the pandemic.

**Conclusion:**

This study illustrates a need for community-engaged COVID-19 vaccine messaging which reflects the nuances of the COVID-19 vaccine and pandemic without oversimplifying information. In highlighting common concerns of the Greater Boston Area which contribute to a lack of confidence in the COVID-19 vaccine, we underscore important considerations for public health and healthcare leadership in the development of initiatives which work to advance health equity.

**Supplementary Information:**

The online version contains supplementary material available at 10.1186/s12889-023-15198-6.

## Background

Research and front-line clinical experiences have made it clear that COVID-19 vaccines provide a strong level of protection against severe disease, hospitalization, and death [1–4] even in the face of waning ability to prevent transmission against new variants [5–7]. Black and Latino communities have been disproportionately impacted by COVID-19 despite the availability of vaccines; vaccine administration data show 42.3% of Black individuals, 53.7% of Latino individuals, and 49.0% of white individuals are fully vaccinated [8]. To date, Latino and Black individuals are hospitalized with COVID-19 at rates of 2.3 and 2.4 times higher than their white counterparts, respectively [9].

Current challenges in vaccine uptake stem from multiple factors including historical exploitation of Black individuals, contemporary experiences of racism, structural barriers to care, and an unfamiliarity with the vaccine development process and inconsistent messaging from public health and government officials [10–13]. Inequities in COVID-19 vaccination rates may stem from historical factors such as the Tuskegee Syphilis Study and contemporary effects of structural racism which negatively impacts on health outcomes, health care access, employment, and housing [10, 11, 14, 15]. Several studies have found that inequities in COVID-19 mortality and vaccine uptake are directly associated with measures of structural racism present in communities of color [16–18]. Further, challenges faced by immigrant communities, such as language barriers, fear of deportation, and insurance coverage concerns also impact inequities in COVID-19 vaccination rates [19].

To overcome mistrust and succeed in both COVID-19 vaccination campaigns and future public health programs, strategies must be developed which: (1) address medical mistrust and; (2) motivate health behavior change among traditionally marginalized communities. During the pandemic, medical centers caring for diverse communities developed culturally and linguistically inclusive approaches to engage their communities. Here we present Boston Medical Center’s (BMC) approach of using a series of community-engaged conversations to elicit and address concerns regarding the COVID-19 vaccine. BMC has played a significant role in vaccinating medically underserved people within the Greater Boston Area [20]. The following paper is an analysis of the resulting themes from these community-engaged conversations, as well as proposed next steps for healthcare leaders and public health professionals.

## Methods

### Study setting

BMC is the largest urban safety-net hospital in New England, providing care to patients regardless of their ability to pay or their immigration status; many of BMC patients rely on Medicare and Medicaid for healthcare coverage [21, 22]. Of patients treated at BMC for COVID-19 between March and May 2020, 44.6% were Black, 30.1% were Hispanic, and 16.4% were experiencing homelessness [23].

### Study sample and data collection

From March 2021 to September 2021, BMC organized a series of nine conversations in partnership with groups such as the NIH “All of Us” Research Study [24]. The events were led by trusted community leaders, such as faith leaders, leaders of local community organizations, local sports celebrities, and healthcare professionals from respective communities, as well as healthcare professionals employed by BMC. The community conversations provided space to address community concerns, answer questions regarding the COVID-19 pandemic, and encourage COVID-19 vaccination. All nine conversations were held via the Zoom audio-video platform; six were simultaneously shared on Facebook Live and two were broadcast on local radio stations. Each of these conversations garnered attendance of between 8 and 122 participants and demographic information for participants was not collected. Recruitment methods for these conversations included print advertisements, organic and paid social media and digital advertisements, and radio advertisements. Participants could write or ask their questions live. Six conversations prioritized specific groups: the Haitian-Creole community, the Cape Verdean community, the Latino community, the Black Christian Faith community, guardians who care for children living with disabilities, and individuals affected by systemic lupus erythematosus. Remaining conversations targeted the general public of the Greater Boston Area. Six conversations were conducted in English, one in Spanish, one in Haitian-Creole, and one in Cape Verdean Creole. Three of the conversations included a short presentation for participants followed by a question-and-answer format; the remaining conversations were strictly conducted in a question-and-answer format. The community conversations were recorded and transcribed verbatim in the respective spoken language by a professional transcription company; if the conversation was not in English, it was then translated into English for data analysis. As these community conversations were hosted on Zoom and participants were able to type questions and comments in the chat, only those questions and comments which were read aloud, and therefore were transcribed from the audio recording, are included in this analysis.

For this study, we accessed those publicly available recordings and employed qualitative methods to characterize and discuss emerging themes. This allowed us to rapidly identify areas for future initiatives to increase vaccination in hard-hit communities.

### Data analysis

To conduct the qualitative analysis, we created a draft codebook based on the Consolidated Framework for Implementation Research (CFIR). The CFIR framework is organized into five domains: Innovation Characteristics, Outer Setting, Inner Setting, Individual Characteristics, and Process [25]. Each domain consists of constructs which influence or impact that domain. While CFIR is traditionally used in implementation research [26], we used CFIR as a decision-making framework to inform implementation of future interventions to increase vaccination. To do this, we modified the CFIR-based codebook to capture elements specific to COVID-19 vaccination. Three study team members (MT, AT, and MLD) first independently reviewed two transcripts and applied the initial CFIR-based codebook to the contents. Reviewers also used emergent coding to identify themes not captured by CFIR constructs and further refine the codebook. To reach consensus among coding, analysts discussed disagreements and came to a resolution. Two team members (MT and AT) used NVivo 12.7.0 (2019) for final coding and analysis.

In light of the need for timely data, we utilized a modified version of rapid analysis methods [27, 28]. First, we created summary templates of each transcript and summarized data from each CFIR construct with space for illustrative quotes from each construct. Second, we aggregated the summaries for each construct across all transcripts in a Thematic Analysis Template. Two analysts (MT and AT) completed the Thematic Analysis Template for all CFIR constructs. The two analysts then met to discuss similarities and differences and refine preliminary themes and illustrative quotes. Third, once agreement was reached, analysts populated the preliminary themes into a spreadsheet matrix and met to generate emerging themes across transcripts and CFIR constructs.

## Results

### Overview of themes

**Fig. 1:**
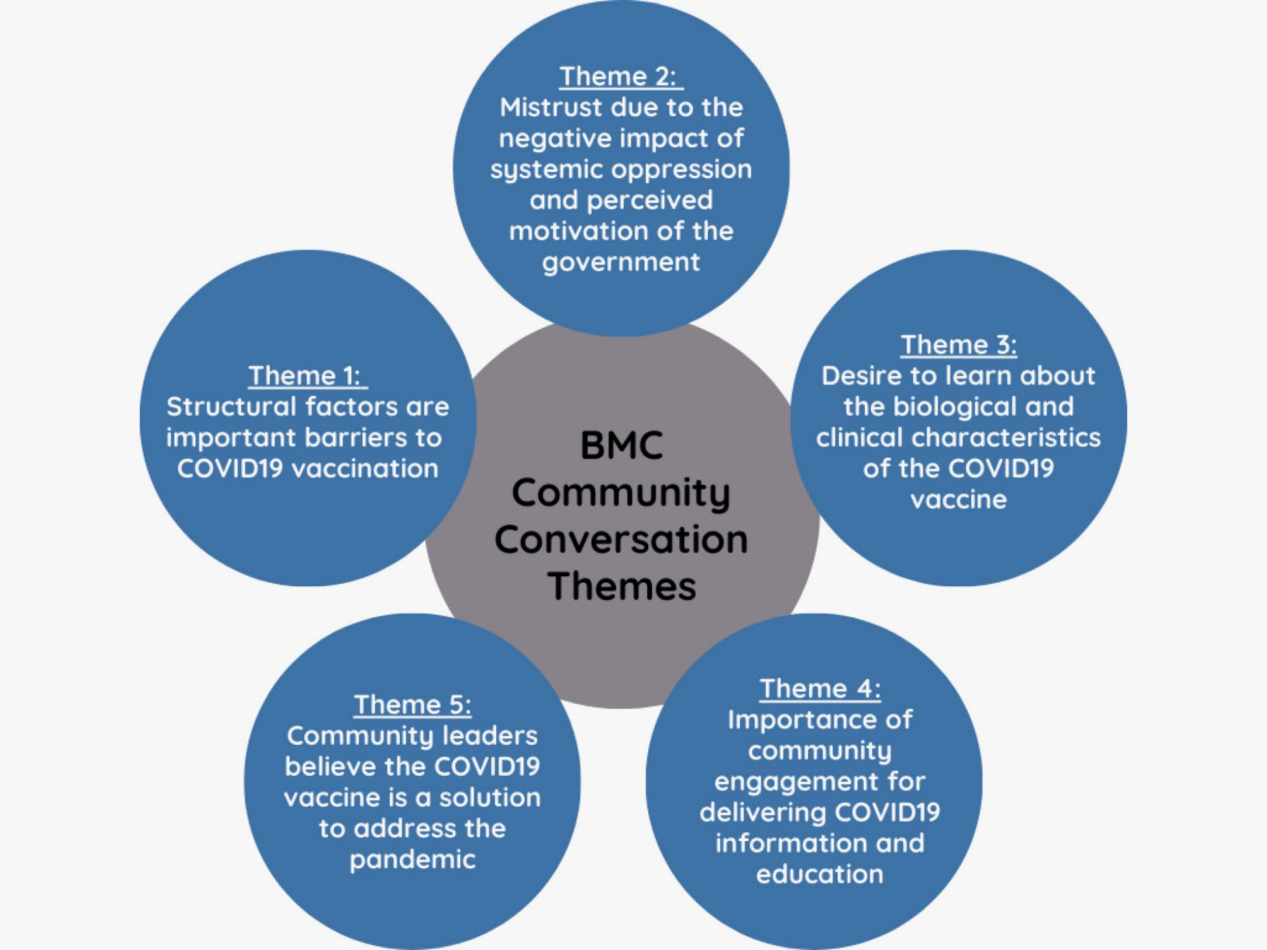
Overview of Themes

We identified five themes that characterized general concerns in the community regarding the COVID-19 vaccine and the type of messaging community leaders believed would encourage vaccination. We describe these themes with illustrative quotes below (see Fig. 1). A table reflecting the presence of each theme across conversations can be found as a supplementary table (Table S1).

### Theme 1: structural factors are important barriers to COVID-19 vaccination

Communities identified multiple structural barriers to receiving the vaccine. Language differences impacted individuals’ ability to understand messaging, schedule appointments, and communicate with healthcare providers. A Cape Verdean leader reflected that, *“Many Cape Verdeans do not speak English, because they arrived recently in the USA. The message does not reach them or if they got it, they don’t understand it very well. They have too many questions.” (Cape Verdean Community Conversation)* Other structural barriers included location of vaccine sites, access to those locations, including access to transportation to those sites, and ease of scheduling an appointment. A leader of the Haitian-Creole community revealed another concern of community members was, *“… will the vaccine really come to us since they started far away? Do we have car access so we can go all the way to [Mass vaccination site set up at football stadium outside of Boston] to get the vaccine?” (Haitian-Creole Community Conversation)*.

This theme relates to the CFIR Domains of Innovation Characteristics, Outer Setting, and Individual Characteristics. Structural barriers to vaccine access are related to the distribution processes of the vaccine (Innovation Characteristics), contextual factors which may facilitate or inhibit an individual’s access to the vaccine (Outer Setting), and individual factors such as differences in language (Individual Characteristics).

### Theme 2: Mistrust due to the negative impact of systemic oppression and perceived motivation of the government

Mistrust originating from the negative impact of systemic oppression and perceived motivation of the government was a barrier to vaccination. Participants expressed deep race-related medical mistrust stemming from historical experiences of medical oppression and exploitation of communities of color. Community leaders from the Haitian-Creole and Cape Verdean communities acknowledged the long history of medical exploitation in communities of color. They also underscored that these historical experiences are at the root of some of the mistrust expressed by their communities towards the medical and scientific communities today: *“…research was made in minority communities without their consent. They did not respect the people’s civil and individual rights. Then the people start to doubt and resist. Since then, people have been questioning the vaccine.” (Cape Verdean Community Conversation)*.

Leaders within the Haitian-Creole community further noted that their community expressed mistrust in vaccine efforts and a concern that the vaccination program could be harmful to Black individuals: *“… since historically in the matter of vaccines, where Blacks still suffer and carry a series of heavy burdens… ‘is this vaccine good for Blacks? is it not a way to eliminate, and reduce Black races?’” (Haitian-Creole Community Conversation)*.

Another component of mistrust was grounded in government involvement in the COVID-19 vaccine development and distribution processes. People expressed a lack of familiarity with vaccine licensing and approval processes. One participant asked through the chat, *“if the vaccine is so safe, why is it not approved by the FDA?” (Black Christian Faith Community Conversation)* Leaders noted that a lack of vaccine confidence was further enhanced by widespread misinformation touting the vaccine as a government tracking device, ability of the vaccine to change one’s DNA, and fear of receiving the vaccine in relation to one’s immigration status. *“…[O]ne question is, I don’t want the government tracking me, none of us do.” (General Public Community Conversation 1)*.

This theme is reflective of the Outer Setting domain of CFIR, as systems of oppression such as systemic racism influence attitudes towards the vaccine.

### Theme 3: Desire to learn more about biological and clinical characteristics of the COVID-19 vaccine as well as practical implication of being vaccinated

During these conversations community leaders reiterated that community members wanted clarification about COVID-19 vaccine components, how they confer protection, and how long protection provided by vaccination would last: *“…how is the vaccine done, what is inside the vaccine?” (Haitian-Creole Community Conversation)* Individuals also wanted more information about the COVID-19 vaccine’s side effects and whether these differed depending on whether someone had or had not been previously infected with COVID-19: *“Is there any difference between those who already got COVID and got the vaccine after several months, and those who never got the virus and get vaccinated, as far as the side effects?” (Systemic Lupus Erythematosus Community Conversation)* Further, individuals were curious how previous infection with COVID-19 affected their need for the vaccine: *“If someone has already had COVID-19, did they still need to get the vaccine and why?” (General Public Community Conversation 1)*.

Regarding the practical implications of the COVID-19 vaccines, community members also questioned the importance of non-pharmaceutical interventions such as masks and social distancing in addition to the COVID-19 vaccine, and whether these public health interventions were interchangeable with vaccines. One community leader recalled a common question within their community: “*Why get a vaccine if we have to wear a mask and why to wear a mask if we are vaccinated?” (Cape Verdean Community Conversation)*.

Theme 3 reflects the knowledge of community members and is therefore related to the Individual Characteristics domain of CFIR.

### Theme 4: community leaders emphasize community engagement for delivering COVID-19 information and education

Community leaders underscored two methods to effectively engage their communities in conversations about the COVID-19 vaccine, namely leveraging existing social networks and using trusted community voices. They believed that public health officials could leverage existing social networks to become an effective vehicle for disseminating COVID-19 vaccine-related information and education. However, community leaders also believed that these networks are underutilized. *“We are trying to reach out because we know that community members have more impact on their neighbors than celebrities or politicians that kind of thing, so I think that people have made an effort, perhaps based on that question, not enough of an effort, so maybe we should double down and try to be better at that and do more at that.” (General Public Community Conversation 2)*.

The second suggested strategy for community engagement by leaders was using trusted community leaders and established community and/or religious organizations to deliver credible information to their communities: *“There are important people, such as [name of Medical Professional 2], a doctor, who has given especially important pieces of advice, we trust him, as well as the elderly.” (Cape Verdean Community Conversation)*.

Theme 4 is primarily related to the Process domain of CFIR due to its emphasis on methods to increase COVID-19 vaccine confidence.

### Theme 5: Community leaders believe that the COVID-19 vaccine is a solution to address the pandemic

Community leaders framed their responses to participants’ concerns about the COVID-19 vaccine by addressing both the social and the health benefits of receiving the COVID-19 vaccine. In terms of the social benefits, the vaccine was seen as allowing for a return to normal community function. They expressed that receiving the vaccine would allow communities to gather and engage in social activities as they did prior to the start of the pandemic: *“We will have the same ability as before of going to shops or restaurants or to the park to watch the Red Sox … the only reason they can [reopen as normal] is because the vaccine exists and because people are getting it. And if you want to be in a place where you can be as before, the vaccine is your best friend.” (Latino Community Conversation)* They also expressed their thoughts that the vaccine was a tool to keep the community healthy during the COVID-19 pandemic. *“When you are vaccinated you develop antibodies. The body is prepared for this little germ, the body is prepared for this virus, if it comes into the body, you have soldiers to fight, to protect the body…” (Haitian-Creole Community Conversation)*.

This theme relates to the Individual Characteristics domain of CFIR, as it largely reflects the priorities of community leaders and how they perceive and approach messaging to increase COVID-19 vaccine confidence.

## Discussion

Our findings highlight the concerns of Greater Boston communities and reflect the beliefs and approaches of local community leaders between March and September of 2021. First, we found that accessibility to the COVID-19 vaccine was limited due to structural barriers like language differences, access to transportation, and concerns regarding immigration status. Additionally, trust in the vaccine was negatively impacted by systemic racism and perceived motivations of the government. Third, there was also interest in understanding the biological and clinical characteristics of the COVID-19 vaccine. Fourth, social networks and community engagement were viewed as essential methods for disseminating COVID-19 information and education. Lastly, community leaders presented the COVID-19 vaccine as a solution to end the pandemic.

We also related these concerns to the CFIR domains to highlight areas of implementation which could benefit from further development to address community concerns. Our first theme which identifies structural barriers points to adapting intervention characteristics within the Innovation Characteristics, Outer Setting, and Individual Characteristics domains of CFIR to help address access to vaccination and increase vaccine confidence. Our second theme highlighting the impact of systemic barriers and perceived government motivations on health inequities allows for a deeper understanding of the current social context within respective communities, or the Outer Setting domain of CFIR, to further inform COVID-19 vaccine initiatives. Our third theme underscores interest in understanding the biological and clinical characteristics of the COVID-19 vaccine and highlights the importance of prioritizing efforts that identify and address knowledge gaps within communities to elevate COVID-19 vaccine confidence; this relates to the Individual Characteristics domain of CFIR. Our fourth theme emphasizes community engagement as a tool to increase vaccine confidence and points to the potential of utilizing processes identified by the communities who are the intended recipients of intervention efforts; this relates to the Process domain of CFIR. Lastly, our fifth theme regarding community leaders’ approach to end the pandemic relates to the Individual Characteristics domain of CFIR, and further understanding of these perceptions may inform future COVID-19 vaccine initiatives that raise vaccine confidence.

Our data are consistent with literature highlighting that structural barriers such as language barriers [19], vaccine location [29], and concerns about immigration status [30] present barriers to vaccination in immigrant communities. Community conversations held in immigrant communities referenced many of these same concerns in relation to the COVID-19 vaccine.

Data also show that historical and current experiences of medical racism continue to drive inequities in vaccine confidence and vaccination rates within US Black communities by contributing to race-related medical mistrust [10, 13, 31, 32]. While research points to structural barriers as prominent drivers of vaccine inequities in immigrant communities, our data suggest that Black immigrant communities may share similar concerns which contribute to lack of vaccine confidence, such as historical experiences of racism.

Uncertainties regarding vaccines have historically played a role in lack of vaccine confidence [33–35]. We found varying levels of knowledge regarding the biological and clinical characteristics of the COVID-19 vaccine in Greater Boston communities, and these uncertainties may contribute to lack of COVID-19 vaccine confidence and, in turn, lower vaccination rates.

Lastly, community engagement can be an effective approach for eliciting drivers of inequities in vaccine uptake and confidence, and have been successful in past COVID-19 initiatives in urban settings [36–39]. Our findings underscore the importance of strong community partnerships and trusted community leaders to advance equity in COVID-19 vaccine confidence and COVID-19 related outcomes.

Despite the continuously shifting landscape of the COVID-19 pandemic, some of our identified themes remain prominent concerns and are supported by national data. Between August of 2020 and April of 2022, concerns centered around vaccine approval processes, side effects, mistrust in the effectiveness of COVID-19 vaccines, mistrust in the government and their role in vaccine development and distribution processes, and more recently the use of masks and mask mandates in public spaces [40–46]. The resonance of our findings with today’s current landscape underscores gaps in public health efforts and areas we should prioritize for the future.

Our results highlight two crucial considerations: (a) structural barriers still present major obstacles to receiving the COVID-19 vaccine and; (b) engaging trusted community leaders is critical for improving confidence in COVID-19 vaccines. To prioritize the needs and concerns of our communities, public health messaging must reflect the nuances of the COVID-19 pandemic and the COVID-19 vaccines. COVID-19 vaccines are still our strongest public health tool, even in the face of new variants and waning immunity [47]. Leveraging a community-engaged approach to develop messaging campaigns that appreciate the nuances of the pandemic in lay language, adapt to the shifting landscape of the pandemic, and provide COVID-19 related care to communities of color can help to refocus community expectations of the COVID-19 vaccine and provide protection guidance without oversimplifying the solution. Community leaders hold trusted positions within communities, and can help to tailor messaging focused on continued protection against severe disease, hospitalization, and death as well as quality of life within the community. Such community-engaged initiatives in urban settings have been successful in the past and can serve as models for future initiatives; key elements of these community partnerships include building trust, centering the voices of community members and trusted community leaders, and focusing on the role of systemic racism in health outcomes and experiences [38, 39].

Community-engaged messaging must be accompanied by concerted efforts to address systemic racism and structural barriers to elevate the health of traditionally marginalized communities. The development of community-level initiatives and messaging campaigns for marginalized communities must explicitly consider not only historical experiences of racism, but also contemporary experiences to contextualize existing health inequities [10, 11, 48]. Efforts at the community level must work in a multi-sectoral fashion and in parallel with efforts at the policy level which address root causes of systemic oppression; efforts include advancing equity in income and wealth, broad support for prioritizing the needs of traditionally marginalized communities, and shifting to models of care that center equity (i.e. trauma- and violence-informed care, culturally safe care, contextually tailored care) [49, 50]. Ultimately, community engagement must occur alongside efforts to address systemic barriers to healthcare; it is necessary to build trust in the medical community in addition to removing the root causes of systemic inequities.

This study has limitations. First, questions and concerns were mainly shared by conversation moderators or conjectured by community leaders rather than asked directly by participants. This method may not capture the nuance of community members’ questions or concerns, and rather may reflect the experiences and perceptions of community leaders. During this early period of the pandemic, community leaders provided an outlet of communication for community members who may have been most impacted by severe social restrictions. Further, due to the nature of the medium in which these community conversations were conducted, the views of individuals who may have not had access to the internet (e.g. lack of funds, older individuals, persons experiencing homelessness or with housing instability) might not be represented in this data set. Although many of the concerns reflected in this manuscript may reflect the experiences and perceptions of community leaders, their role in these conversations and connections with their community provided a forum for community voices that would have otherwise been unheard. Second, traditional rapid analysis methods typically include a step where analysts rate each summary to capture the effect (positive or negative) and strength (weak or strong) on the desired behavior outcome [27]. We elected not to assign summary ratings due to the largely question and answer format of these conversations, which did not provide enough context to confidently rate each construct summary and determine its effect on behavior. This may limit our analytic ability to infer behavior and compare our findings with other similar approaches [27]. Lastly, these results are reflective of traditionally marginalized communities living in an urban setting of the Greater-Boston Area. Therefore, our results may not be generalizable to traditionally marginalized communities in suburban or rural settings.

## Conclusion

In conclusion, our findings highlight common concerns which contribute to lack of vaccine confidence in the Greater Boston Area. Engaging trusted community leaders to better understand community needs related to the COVID-19 vaccine can help prioritize and inform initiatives which increase vaccination rates in marginalized communities, such as Black and Latino communities, and work towards advancing health equity.

## Electronic supplementary material

Below is the link to the electronic supplementary material.

Supplementary Material 1

## Data Availability

The data that support the findings of this study are available upon request from the Institutional Review Board at Boston University Medical Campus and Boston Medical Center at medirb@bu.edu.

## Abbreviations

BMC: Boston Medical Center
CFIR: Consolidated Framework for Implementation Research
