# Supplementary figures and images for: “Community members have more impact on their neighbors than celebrities”: leveraging community partnerships to build COVID-19 vaccine confidence
Source: BMC Public Health. 2023 Feb 16;23:350. doi: 10.1186/s12889-023-15198-6 (PMC9933023; doi:10.1186/s12889-023-15198-6)

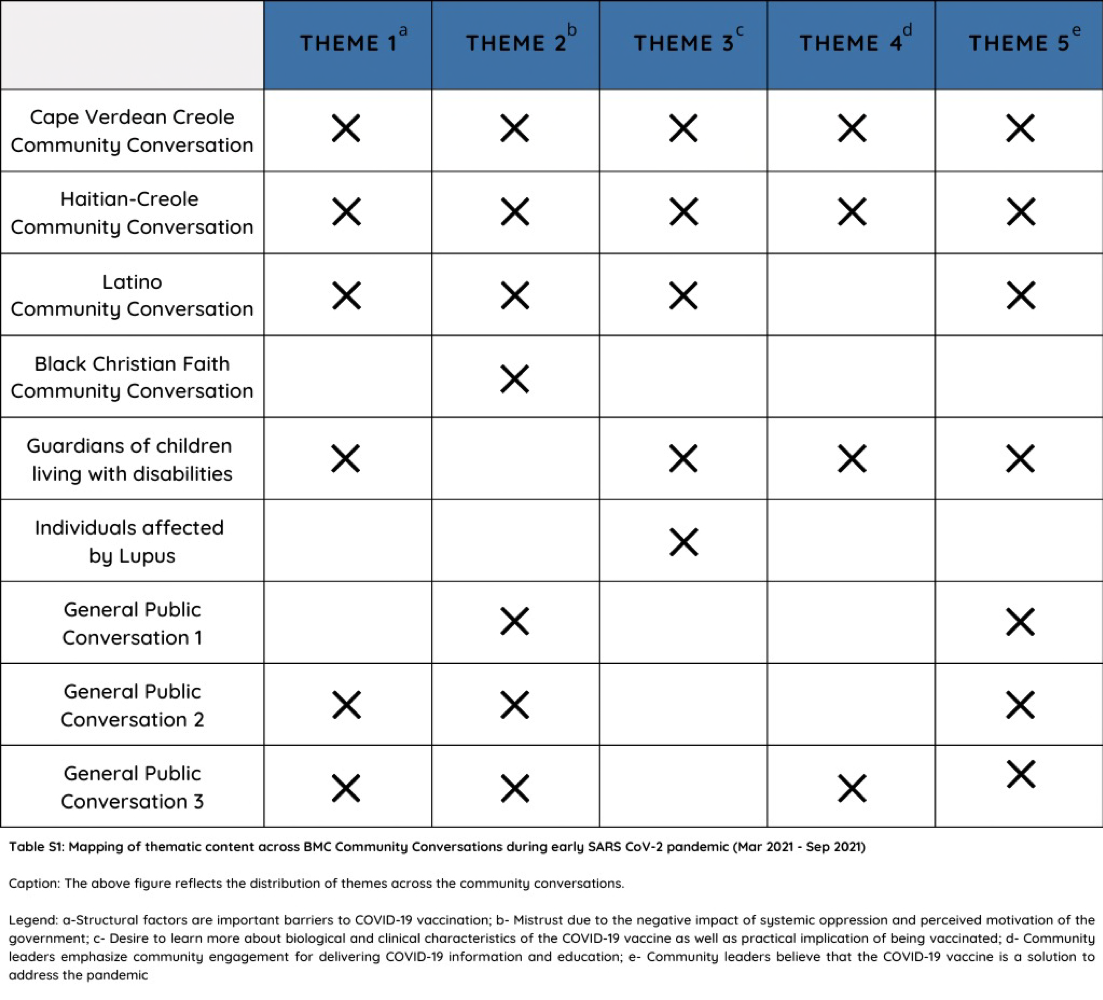

Supplement: Supplementary file 1 — Supplementary Material 1 [file 12889_2023_15198_MOESM1_ESM.png]
